# Impact of prognostic nutritional index change on prognosis after colorectal cancer surgery under propofol or sevoflurane anesthesia

**DOI:** 10.1186/s12871-023-02308-5

**Published:** 2024-01-03

**Authors:** Longtang Zhang, Chong Liu, Qiang Yan, Xiaoli Cai

**Affiliations:** 1 Department of Anesthesiology, the Fifth Affiliated Hospital of Xinjiang Medical University, No. 118 Henan West Road, Xinshi District, Urumqi City, Xinjiang China; 2 Department of Anesthesiology, Xinjiang Uygur Autonomous Region People’s Hospital, No. 91 Tianchi Road, Tianshan District, Urumqi City, Xinjiang China

**Keywords:** Colorectal cancer, Prognostic nutritional index, Propofol-based anesthesia, Sevoflurane-based anesthesia, Overall survival, Progression free survival

## Abstract

**Background:**

The alteration of the prognostic nutritional index (PNI) or the utilization of distinct anesthesia strategies has been linked to the prognosis of various cancer types, but the existing evidence is limited and inconclusive, particularly for colorectal cancer (CRC). Our objective was to evaluate the association between PNI change and progression free survival (PFS) and overall survival (OS) in patients treated with CRC surgery after propofol-based or sevoflurane-based anesthesia.

**Methods:**

We conducted a retrospective analysis of 414 patients with CRC who underwent surgical resection. Among them, 165 patients received propofol-based total intravenous anesthesia (TIVA-P), while 249 patients received sevoflurane-based inhalation anesthesia (IA-S). The PNI change (ΔPNI) was calculated by subtracting the pre-surgery PNI from the post-surgery PNI, and patients were categorized into high (≥ -2.25) and low (< -2.25) ΔPNI groups. Univariate and multivariate analyses were employed to evaluate the effects of the two anesthesia methods, ΔPNI, and their potential interaction on PFS and OS.

**Results:**

The median duration of follow-up was 35.9 months (interquartile range: 18–60 months). The five-year OS rates were 63.0% in the TIVA-P group and 59.8% in the IA-S group (hazard ratio [HR]: 0.96; 95% confidence interval [CI]: 0.70–1.35; *p* = 0.864), while the five-year PFS rates were 55.8% and 51.0% (HR: 0.92; 95% CI: 0.68–1.26; *p* = 0.614), respectively. In comparison to patients in the low ΔPNI group, those in the high ΔPNI group exhibited a favorable association with both OS (HR: 0.57; 95% CI: 0.40–0.76; *p* < 0.001) and PFS (HR: 0.58; 95% CI: 0.43–0.79; *p* < 0.001). Stratified analysis based on ΔPNI revealed significant protective effects in the propofol-treated participants within the high ΔPNI group, whereas such effects were not observed in the low ΔPNI group, for both OS (p for interaction = 0.004) and PFS (p for interaction = 0.024).

**Conclusions:**

Our data revealed that among patients who underwent CRC surgery, those treated with TIVA-P exhibited superior survival outcomes compared to those who received IA-S, particularly among individuals with a high degree of PNI change.

## Background

According to the Global Cancer Statistics 2020 report published by the International Agency for Research on Cancer, colorectal cancer (CRC) has emerged as the second leading cause of cancer-related mortality and the third most prevalent cancer worldwide [1]. It is estimated that over 1.9 million new cases and 935,000 deaths occurred in 2020, accounting for approximately one-tenth of all cancer cases and deaths [1]. Currently, surgical resection stands as the established and primary treatment modality for CRC [2]. Despite significant advancements in surgical and medical techniques, the prognosis for patients with CRC still requires improvement [3]. Apart from surgical techniques, numerous factors preoperatively, intraoperatively, or during the postoperative period have been proposed to be associated with the prognosis of CRC [4].

Surgical interventions elicit an inflammatory response attributed to tissue damage [5]. Within the perioperative period, various factors, including immune and nutritional status, have been proposed to contribute to a micro-metastatic process, consequently leading to unfavorable long-term oncological outcomes [6]. The prognostic nutritional index (PNI), which incorporates serum albumin levels and total lymphocyte count in peripheral blood, represents a widely employed metric for evaluating the nutritional and immunological statuses of cancer patients [7]. The alteration in PNI before and after surgery reflects the dynamic changes in patients' immune responses and nutritional statuses [8]. Notably, the baseline prognostic nutritional index and changes in pre-treatment body mass index have been associated with the response to immunotherapy in patients with advanced cancer [9–11]. However, no studies have investigated the association between preoperative PNI changes and patient outcomes following CRC surgery.

Diverse anesthesia techniques can potentially influence the oncological prognosis by modulating innate and cellular immunity and activating adrenergic-inflammatory pathways [12]. Volatile inhalational agents and intravenous anesthetic agents are commonly employed for anesthesia maintenance during cancer surgeries [13]. It has been proposed that volatile inhalational agents, such as sevoflurane, might enhance cancer cell activity by suppressing immune cell function [14]. Conversely, intravenous anesthetic agents, such as propofol, possess anti-inflammatory and antioxidative properties that could potentially mitigate immune suppression [15, 16]. Several studies have compared the overall survival (OS) or progression-free survival (PFS) of cancer patients undergoing inhalational anesthesia versus total intravenous anesthesia, but the results have been inconclusive [17, 18]. A meta-analysis involving patients undergoing diverse cancer surgeries indicated that total intravenous anesthesia was not associated with improved recurrence-free survival but exhibited improved OS compared to volatile anesthesia [12]. Furthermore, it remains unknown whether these effects could be modified by the inflammatory status.

Consequently, we undertook a retrospective cohort study to compare the OS and PFS between volatile anesthesia and total intravenous anesthesia, as well as the impact of PNI change, and their combined effects. The primary objective of this study was to assess the relationship between distinct anesthesia techniques and nutritional status and the prognosis of cancer in patients undergoing CRC surgery.

## Materials and methods

### Study population

This retrospective cohort study received approval from the Ethical Review Committees of the Fifth Affiliated Hospital of Xinjiang Medical University. Informed consent was waived due to the retrospective nature of the study. The investigation was conducted in compliance with the ethical standards outlined in the Declaration of Helsinki (1964) and its subsequent amendments. The medical records of all patients who underwent CRC surgery at the Fifth Affiliated Hospital of Xinjiang Medical University between January 2016 and December 2021 were retrospectively reviewed.

All patients underwent radical resection in accordance with the diagnostic and treatment guidelines for CRC established by the Chinese Society of Clinical Oncology (CSCO) in 2018 [19]. The inclusion criteria for this study were as follows: (1) patients aged 18 years or older, (2) patients who underwent curative-intent pancreatic resection, (3) patients with comprehensive clinical examination and histopathologically confirmed results, (4) patients who experienced satisfactory postoperative recovery and were discharged, and (5) patients who could be followed up for a minimum of 3 months. A total of 755 CRC patients who had undergone radical surgery for CRC were initially identified through the electronic medical record system.

**Fig. 1:**
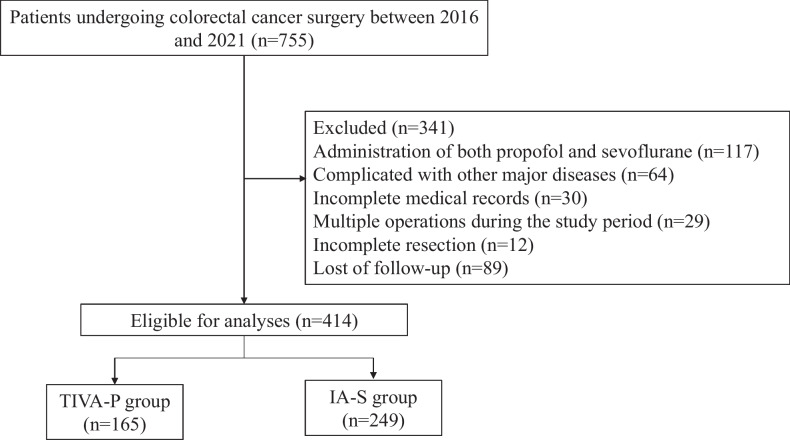
Flow chart of study patient selection

However, several patients were subsequently excluded for the following reasons: (1) patients who received both propofol and sevoflurane anesthesia (*n* = 117), (2) patients with coexisting major diseases (e.g., heart diseases and other cancers) (*n* = 64), (3) patients with incomplete baseline information (*n* = 30), (4) patients who underwent multiple operations during the study period (*n* = 29), (5) patients with incomplete resection (*n* = 12), and (6) patients lost to follow-up (*n* = 89). Ultimately, a total of 414 CRC patients were included in the analysis. The flowchart depicting the patient selection process is presented in Fig. 1.

### Data collection

Patients' demographic characteristics and clinical information were systematically retrieved from the electronic inpatient system. The baseline information encompassed variables such as age, gender, height, weight, smoking and alcohol consumption status, presence of significant comorbidities (including major cardiac conditions and other malignancies), surgical history, specific surgical techniques employed, anesthesia modalities utilized, preoperative and postoperative serum albumin levels, blood lymphocyte counts, tumor location and size, administration of neoadjuvant and adjuvant therapies, duration of surgery and the days of hospital stay, as well as the presence of lymphatic or perineural invasions. Pathological data, including the histological type of CRC and tumor stage, were extracted from the comprehensive records of histopathological evaluations of tissue specimens.

### Anesthesia methods

All patients underwent preoperative central venous puncture and catheterization for hemodynamic monitoring. Vital signs including blood pressure, heart rate, electrocardiogram, oxygen saturation, end-tidal carbon dioxide, central venous pressure, bispectral index (BIS), urine output, and body temperature were routinely monitored. Patients were assigned to the propofol group or the sevoflurane group was based on the anesthesiologist’s preference. In the propofol group, patients received total intravenous general anesthesia. The target-controlled infusion (TCI) concentration of propofol was maintained at 4 mg/L, sufentanil citrate infusion at 0.3–0.5 μg/kg, cisatracurium besilate injection at 0.15–0.20 mg/kg, and maintained with remifentanil hydrochloride infusion at 4–6 ng/L. The sevoflurane group underwent combined intravenous and inhalational general anesthesia. The induction methods involved administering sufentanil at a dose of 0.3–0.5 μg/kg, followed by priming the anesthesia circuit with sevoflurane. Subsequently, a mask was placed on the patient's face, and the patient was instructed to take deep breaths. An 8% concentration of sevoflurane was administered with an oxygen flow rate of 6 L/min. Once the eyelash reflex disappeared, cis-atracurium was given at a dose of 0.15–0.20 mg/kg and maintained throughout the operation. The exhaled concentration of sevoflurane was maintained at 0.8–1.2 minimum alveolar concentration (MAC), and the target concentration of remifentanil TCI was maintained at 4–6 ng/L.

The BIS value was continuously monitored during the surgical procedure in both groups, and it was maintained within the range of 40–60. The dosage of various medications was adjusted based on the anesthesia requirements and BIS value, with the addition of muscle relaxants as needed for the surgery. Patient's body temperature was diligently maintained and monitored using an inflatable warming system (BairHugger, 3 M Company, United States). Heating was discontinued if the temperature exceeded 37 °C, while efforts were made to keep it above 36 °C. Intraoperative fluid replacement and administration of vasoactive drugs were guided by arterial blood pressure and central venous pressure values, ensuring a minimum urine output of at least 0.5 ml/kg/h.

### Prognostic nutritional index calculation

PNI was determined using the following mathematical expression: PNI = albumin (g/L) + 5 × total lymphocyte count (10^9^/L) [20]. Laboratory data for serum albumin and lymphocyte count were extracted at the time of diagnosis and the last recorded data after the surgical procedure prior to discharge, allowing for the calculation of both pre-surgery PNI and post-surgery PNI. The PNI change (ΔPNI) was defined as the difference between the pre-surgery PNI and the post-surgery PNI (ΔPNI = pre-surgery PNI—post-surgery PNI).

### Assessment and follow-up

Outcome measures were obtained by gathering pertinent data from the hospital records or through communication with the patient's family during a telephone-based follow-up. The study concluded on December 31^st^, 2021. The primary endpoints consisted of OS and PFS. OS was defined as the duration from the surgical procedure to the occurrence of death from any cause or until the last follow-up date. PFS was defined as the duration from the surgery to the initial documented evidence of disease progression, occurrence of death from any cause, or until the last follow-up date.

### Statistical analysis

Continuous variables were presented as mean ± standard deviation (SD), and the independent samples t-test was employed to compare intergroup differences between the Total Intravenous Anesthesia-Propofol (TIVA-P) group and the Inhalational Anesthesia-Sevoflurane (IA-S) group. Frequency variables were expressed as n (%) and analyzed using a chi-square test.

Kaplan–Meier survival curves were constructed based on group differences and analyzed using the log-rank test. Univariate and multivariate analyses were performed using the Cox proportional hazards regression model. The hazard ratio (HR) and 95% confidence interval (CI) were used to estimate relative risks. The following covariates were adjusted in the models: age, gender, body mass index (BMI), smoking or alcohol consumption status, histological type, American Society of Anesthesiologists (ASA) stage, type of surgery, tumor location, presence of lymphatic or perineural invasion, and neoadjuvant or adjuvant therapy.

To explore the joint effects of PNI change with propofol-based or sevoflurane-based anesthesia on the prognosis of patients following CRC surgery, we incorporated product terms into the Cox regression models to evaluate the interactions.

The data were analyzed using SPSS 25.0 (SPSS Inc., Armonk, New York) and R 3.6.3 (http://www.r-project.org/). All statistical tests were two-sided, and a significance level of *P* < 0.05 was applied to determine statistical significance.

## Results

### Baseline characteristics

Table 1 illustrates the demographic and clinical characteristics of the study participants. Patients in the TIVA-P group exhibited a statistically significant higher mean age compared to those in the IA-S group (66.7 ± 12.4 years vs. 64.2 ± 11.5 years, *P* = 0.039). However, no significant differences were observed between the cases and controls regarding age distribution, gender distribution, BMI, smoking or alcohol consumption status, histological type, ASA stage, type of surgical procedure, tumor location, lymphatic or perineural invasion, neoadjuvant or adjuvant therapy, duration of surgery and the days of hospital stay (all *P* > 0.05).

**Table 1:** Baseline characteristics of included patients

	TIVA-P group (*n* = 165)	IA-S group (*n* = 249)	*P*
Age (yrs)	66.7 ± 12.4	64.2 ± 11.5	**0.039**
Gender, n(%)			0.365
Male	81 (49.1)	110 (44.2)	
Female	84 (50.9)	138 (55.4)	
BMI (kg/m^2^)	24.7 ± 3.98	24.9 ± 2.24	0.557
Smoking status, n(%)			0.350
Current or ever smoking	44 (26.7)	56 (22.5)	
Never smoking	121 (73.3)	193 (77.5)	
Alcohol drinking status, n(%)			0.205
Current or ever drinking	47 (28.5)	57 (22.9)	
Never drinking	118 (71.5)	192 (77.1)	
Histological type, n(%)			0.897
Adenocarcinoma	134 (81.2)	204 (81.9)	
Special types of cancer	31 (18.8)	45 (18.1)	
ASA, n(%)			0.605
II	137 (83)	201 (80.7)	
III	28 (17)	48 (19.3)	
Type of operation, n(%)			0.756
Laparoscopy	147 (89.1)	218 (87.6)	
Laparotomy	18 (10.9)	31 (12.4)	
Tumor location, n(%)			0.718
Rectum	126 (76.4)	195 (78.3)	
Colon	39 (23.6)	54 (21.7)	
Lymphatic invasion, n(%)	46 (27.9)	71 (28.5)	0.912
Perineural invasion, n(%)	70 (42.4)	108 (43.4)	0.919
Neoadjuvant therapy, n(%)	19 (11.5)	32 (12.9)	0.761
Adjuvant therapy, n(%)	105 (63.6)	165 (66.3)	0.599
Pre-surgery PNI	48.5 ± 6.32	48.8 ± 6.55	0.641
Post-surgery PNI	46.8 ± 6.14	45.5 ± 6.32	**0.037**
ΔPNI	-1.72 ± 3.52	-5.32 ± 8.76	** < 0.001**

*Abbreviations*: *BMI* body mass index, *PNI* prognostic nutritional index

Regarding the PNI, the preoperative PNI exhibited comparable values between the TIVA-P group and the IA-S group (48.5 ± 6.32 vs. 48.8 ± 6.55, *P* = 0.641). However, postoperative PNI and ΔPNI showed significant differences between the two groups, with the TIVA-P group demonstrating higher postoperative PNI values (46.8 ± 6.14 vs. 45.5 ± 6.32, *P* = 0.037) and a smaller decrease in PNI (ΔPNI) compared to the IA-S group (-1.72 ± 3.52 vs. -5.32 ± 8.76, *P* < 0.001) (Table 1).

### Associations of ΔPNI or propofol-based or sevoflurane-based anesthesia on prognosis of patients after CRC surgery

Throughout the follow-up period (median 35.9 months, interquartile range: 18–60 months), a total of 897 cases of disease progression were observed, which consisted of 161 deaths and 34 cases of recurrence or metastasis. The five-year OS rates were 63.0% in the TIVA-P group and 59.8% in the IA-S group, while the five-year PFS rates were 55.8% and 51.0% respectively.

**Fig. 2:**
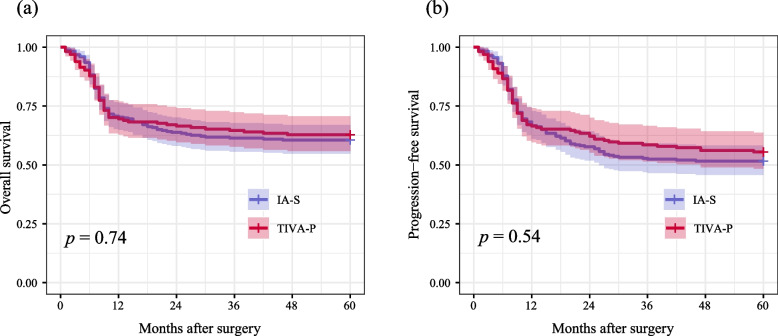
**a** Overall survival curves from the date of surgery by anesthesia type; **b** Progression-free survival curves from the date of surgery by anesthesia type (red line: propofol-based anesthesia, blue line: sevoflurane-based anesthesia)

**Fig. 3:**
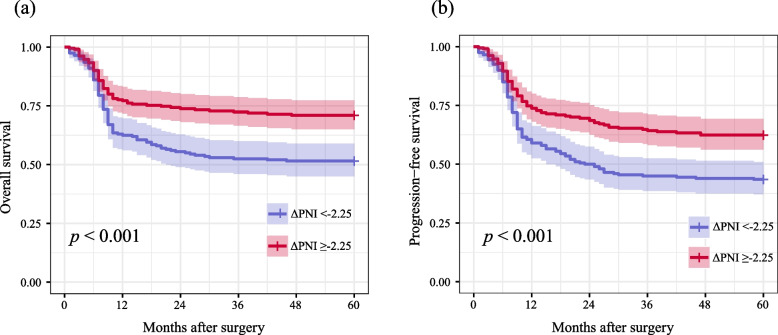
**a** Overall survival curves from the date of surgery by prognostic nutritional index change (ΔPNI); **b** Progression-free survival curves from the date of surgery by ΔPNI (red line: ΔPNI ≥ -2.25, blue line: ΔPNI < -2.25)

Table 2 illustrates the correlations between PNI change or anesthesia methods and the prognosis of patients undergoing CRC surgery. After adjusting for potential confounding factors, no significant differences were observed in OS or PFS between patients in the IA-S group and the TIVA-P group (HR for OS: 0.96; 95% CI: 0.70–1.35; *P* = 0.864) and (HR for PFS: 0.92; 95% CI: 0.68–1.26; *P* = 0.614). Conversely, when comparing the low ΔPNI group (< -2.25) to the high ΔPNI group (≥ -2.25), the low ΔPNI group was associated with superior OS (HR: 0.57; 95% CI: 0.40–0.76; *P* < 0.001) and PFS (HR: 0.58; 95% CI: 0.43–0.79; *P* < 0.001). The Kaplan–Meier survival curves for both PNI change (Fig. 2) and anesthesia methods (Fig. 3) also exhibited similar trends.

**Table 2:** Cox proportional hazards regression analysis of oncologic outcomes: univariable and multivariable models

	Univariate analysis		Multivariate analysis	
	HR (95% CI)	*P*	HR (95% CI)	*P*
Propofol vs. sevoflurane				
Overall survival	0.95 (0.69, 1.31)	0.742	0.96 (0.70, 1.35)	0.864
Progression free survival	0.91 (0.68, 1.22)	0.547	1.92 (0.68, 1.26)	0.614
Preoperative PNI (≥ 48.6 vs. < 48.6)				
Overall survival	0.50 (0.35, 0.70)	< 0.001	0.46 (0.30, 0.68)	< 0.001
Disease free survival	0.54 (0.39, 0.74)	< 0.001	0.52 (0.37, 0.72)	< 0.001
ΔPNI (< -2.25 vs. ≥ -2.25)				
Overall survival	0.54 (0.39, 0.74)	< 0.001	0.57 (0.40, 0.76)	< 0.001
Disease free survival	0.57 (0.43, 0.77)	< 0.001	0.58 (0.43, 0.79)	< 0.001

*Abbreviations*: *HR* hazard ratio, *CI* confidence interval, *PNI* prognostic nutritional index

### Interaction of ΔPNI and propofol-based or sevoflurane-based anesthesia on prognosis of patients after CRC surgery

We conducted additional analysis to examine the modifying effects of PNI change on the relationships between propofol-based or sevoflurane-based anesthesia and the prognosis of patients following CRC surgery. As presented in Table 3, the TIVA-P group exhibited improved OS and PFS compared to the IA-S group. However, these improvements were only observed in patients with a PNI change of < -2.25. The *p*-values for interaction were 0.004 for OS and 0.024 for PFS.

**Table 3:** Cox proportional hazards regression analysis of long-term oncologic outcomes for sevoflurane-based versus propofol-based anesthesia stratified by PNI change

	Overall survival			Progression free survival		
	HR (95% CI)	*P*	P for interaction	HR (95% CI)	*P*	*P* for interaction
ΔPNI			**0.004**			**0.024**
< -2.25	0.46 (0.26, 0.81)	0.007		0.61 (0.39, 0.98)	0.039	
≥ -2.25	1.41 (0.84, 2.38)	0.198		1.32 (0.82, 2.12)	0.251	

*Abbreviations*: *HR* hazard ratio, *CI* confidence interval, *PNI* prognostic nutritional index

## Discussion

In the present study, we conducted a retrospective cohort study to evaluate the impact of PNI change and two anesthesia modalities (propofol-based or sevoflurane-based) on the prognosis following CRC surgery in a Chinese population. Our findings revealed that PNI change, rather than anesthesia methods, exhibited an independent association with an unfavorable CRC prognosis. Moreover, we observed that the beneficial effects of propofol-based anesthesia on the prognosis were exclusively evident among patients with a substantial decline in PNI.

The majority of studies have focused on examining the impact of preoperative PNI on the prognosis of patients with CRC and other malignancies. For instance, Morales et al. [21] conducted a retrospective cohort study in patients with CRC and reported a significant association between PNI and overall survival, suggesting that PNI serves as an independent prognostic factor in CRC patients. Similarly, Tokunaga et al. [22] conducted another retrospective study and found that low preoperative PNI was an independent risk factor for poor overall survival. Furthermore, a systematic review and meta-analysis conducted by Sun et al. [23] demonstrated that a high preoperative PNI was associated with significantly improved overall survival among Asian CRC patients. Consistent with these findings, our study also observed a beneficial effect of preoperative PNI on CRC prognosis.

Limited research has been conducted to investigate the impact of PNI changes on cancer outcomes, particularly in the context of CRC. Kim et al. [8] retrospectively analyzed 107 patients with pancreatic ductal adenocarcinoma who underwent neoadjuvant chemotherapy followed by surgical resection and found that a low PNI change (< -1.94) was an independent risk factor for overall survival (hazard ratio, 3.516; 95% confidence interval, 1.885–6.558; *p* < 0.001), although no significant association was observed with disease-free survival (*p* > 0.05). Our study, for the first time, assessed the impact of PNI change on the prognosis of CRC patients and demonstrated that a lower PNI change was associated with both poorer overall survival and progression-free survival. Preoperative PNI serves as an indicator of the patient's nutritional status prior to surgery, while a low PNI change signifies a significant deterioration in the patient's condition. Conversely, a high PNI change may indicate minimal deterioration or even improvement in the patient's nutritional status [8]. Thus, these findings underscore the importance of considering changes in nutritional status during surgery as a valuable predictor of postoperative prognosis for patients with CRC.

Regarding the impact of two anesthetic agents, namely propofol-based or sevoflurane-based anesthesia, on the prognosis of CRC and other types of cancer, previous studies have yielded conflicting results. For instance, a meta-analysis suggested that total intravenous anesthesia, which includes the use of propofol, may lead to lower mortality rates compared to inhalational anesthesia [24]. Additionally, a retrospective study demonstrated that propofol-based total intravenous anesthesia was associated with improved survival in patients with gastric cancer [25]. Conversely, a study conducted by Makito et al. [13] suggested a limited association between the type of anesthesia and improved OS in patients undergoing surgery for digestive cancers. These inconsistent findings highlight the need for further research in this area. In our study, we did not observe any significant effects of anesthesia methods on the prognosis of CRC.

Furthermore, we observed an interaction effect between changes in PNI and anesthesia on the prognosis of patients with CRC in our study. However, the improved OS and PFS were only evident among patients with a significant decrease in PNI when undergoing propofol-based total intravenous anesthesia. PNI serves as an evaluative index for assessing nutritional and immunological status. Patients with a low PNI may exhibit compromised nutritional status and reduced systemic immunity, which can impact prognosis through local immune responses [26]. Furthermore, the surgical procedure itself activates neural and inflammatory signaling pathways, which are associated with immune dysfunction and cancer development [27]. Therefore, the combined effects of deteriorating nutritional status, surgical trauma, and anesthesia methods on immune mechanisms may contribute to the more pronounced protective effect of propofol-based anesthesia.

Previous evidence has demonstrated that anesthetic medications can modulate various receptor targets on immune cells, highlighting the potential role of anesthetic drugs in immunological processes [28]. Anesthetic agents exert immunological effects on cytotoxic T cells, T helper cells, monocyte macrophages, and other immune cell populations [29]. Studies have suggested that propofol can reduce the expression of tumorigenic growth factors, including hypoxia-inducible factors (HIFs) [30]. Huang et al. [30] found that propofol prevented HIF-1α activation induced by isoflurane and inhibited malignant activities of cancer cells. Furthermore, an in vivo experiment utilizing a pulmonary metastasis model in mice reported that continuous infusion of propofol suppressed pulmonary metastasis, indicating its in vivo anti-invasive action through modulation of Rho A [31].

While this study provides valuable insights into the prognostic implications of the combined effects of PNI changes and anesthesia methods after CRC surgery, it is important to acknowledge certain limitations. Firstly, the study design was retrospective and conducted at a single center. Secondly, the sample size was relatively small, and the lack of validation using an independent cohort introduces the potential for selection bias. Therefore, collaboration with other centers is essential to validate the broader applicability of the current findings in future research. Additionally, the absence of information regarding socioeconomic status, which is known to influence prognosis, is a limitation. However, given that socioeconomic status is associated with educational level and income, which are captured by clinicopathological characteristics, controlling for these variables in the statistical models would mitigate confounding effects to a significant extent. Consequently, future studies with larger sample sizes, multi-center collaboration, and a more comprehensive analysis of exposure factors are warranted to further elucidate the differential prognostic value of PNI changes and anesthesia methods in patients undergoing CRC surgery.

## Conclusions

This study conducted the initial investigation into the interplay between PNI change and the choice of anesthesia (propofol or sevoflurane) on the prognosis following CRC surgery. The results indicate that among CRC surgery patients who experienced a substantial reduction in PNI, those who received propofol-based anesthesia demonstrated a reduced risk of disease progression compared to those who received sevoflurane-based anesthesia. These findings offer potential prognostic indicators and suggest a potential anesthesia protocol for CRC surgery. Further prospective cohort studies and well-designed clinical trials are invited to confirmed the findings.

## Data Availability

The data to support the results of this study is available from the corresponding author on reasonable request.

## Abbreviations

BIS: Bispectral index
CI: Confidence interval
CRC: Colorectal cancer
CSCO: Chinese Society of Clinical Oncology
HR: Hazard ratio
IA-S: Sevoflurane-based inhalation anesthesia
OS: Overall survival
PFS: Progression free survival
PNI: Prognostic nutritional index
TIVA-P: Propofol-based total intravenous anesthesia
ΔPNI: PNI change
